# Correction: Molecular basis of sulfolactate synthesis by sulfolactaldehyde dehydrogenase from *Rhizobium leguminosarum*

**DOI:** 10.1039/d3sc90238b

**Published:** 2023-12-12

**Authors:** Jinling Li, Mahima Sharma, Richard Meek, Amani Alhifthi, Zachary Armstrong, Niccolay Madiedo Soler, Mihwa Lee, Ethan D. Goddard-Borger, James N. Blaza, Gideon J. Davies, Spencer J. Williams

**Affiliations:** a School of Chemistry and Bio21 Molecular Science and Biotechnology Institute, University of Melbourne Parkville Victoria 3010 Australia sjwill@unimelb.edu.au; b York Structural Biology Laboratory, Department of Chemistry, University of York York YO10 5DD UK gideon.davies@york.ac.uk; c Chemistry Department, Faculty of Science (Female Section), Jazan University Jazan 82621 Saudi Arabia; d ACRF Chemical Biology Division, The Walter and Eliza Hall Institute of Medical Research Parkville Victoria 3010 Australia; e Department of Medical Biology, University of Melbourne Parkville Victoria 3010 Australia

## Abstract

Correction for ‘Molecular basis of sulfolactate synthesis by sulfolactaldehyde dehydrogenase from *Rhizobium leguminosarum*’ by Jinling Li *et al.*, *Chem. Sci.*, 2023, **14**, 11429–11440, https://doi.org/10.1039/D3SC01594G.

The authors note that the stereochemistry of several compounds in Fig. 1 were incorrectly drawn. The corrected Fig. 1 and amended figure legend are provided here.

**Fig. 1:**
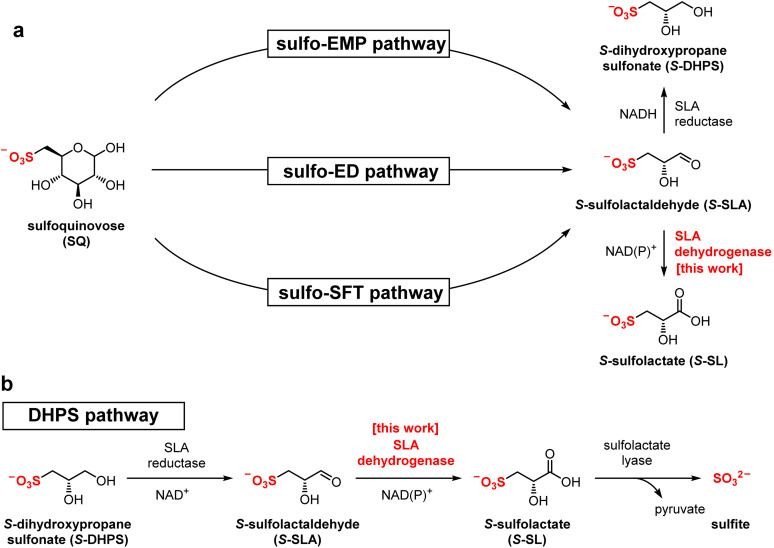
(a) Formation of *S*-sulfolactate (*S*-SL) and *S*-dihydroxypropanesulfonate (*S*-DHPS) through the pathways of sulfoglycolysis from sulfoquinovose (SQ). (b) Formation and degradation of *S*-SL by catabolism of DHPS.

The Royal Society of Chemistry apologises for these errors and any consequent inconvenience to authors and readers.

## Supplementary Material

